# Cathepsin G—Not Only Inflammation: The Immune Protease Can Regulate Normal Physiological Processes

**DOI:** 10.3389/fimmu.2020.00411

**Published:** 2020-03-03

**Authors:** Tatyana S. Zamolodchikova, Svetlana M. Tolpygo, Elena V. Svirshchevskaya

**Affiliations:** ^1^Physiology of Motivation Laboratory, P. K. Anokhin Institute of Normal Physiology, Moscow, Russia; ^2^Immunology Department, Shemyakin-Ovchinnikov Institute of Bioorganic Chemistry RAS, Moscow, Russia

**Keywords:** cathepsin G, Paneth cells, inflammation, homeostasis, duodenal mucosa, intestinal glands

## Introduction

Cathepsin G (CathG) is a serine protease that controls the functional state of immune cells and is traditionally considered to be one of the effectors of inflammation (1). The substrate specificity of human CathG is dual, combining trypsin and chymotrypsin-like properties, thus, CathG can cleave peptide bonds formed by the carboxyl group of both positively charged (arginine, lysine) and aromatic (phenylalanine, leucine, tyrosine) amino acid residues (2). CathG gene is mainly expressed in polymorphonuclear neutrophils which are the first cells recruited to inflammatory sites (3). As a component of neutrophil proteolytic machinery CathG regulates the inflammatory responses by stimulating the production of cytokines and chemokines, which are responsible for the activation and mobilization of immune cells to the site of pathogen and/or tissue damage (1, 3). CathG activates metalloproteases and cleaves extracellular matrix proteins, contributing to neutrophil migration (4). CathG-specific hydrolysis of receptors, enzymes, cytokines, and other biologically active peptides leads to a modulation of chemotaxis and intercellular interactions, activation of the local renin-angiotensin system (RAS) of granulocytes, initiation of apoptosis, and many other processes (5–7). CathG is synthesized by immunocytes both in a secretory form (neutrophils, mast cells), and in a form of intracellular protein (antigen-presenting cells) (8–10). CathG is also found in some non-myeloid cells, such as endothelial and smooth muscle cells, brain astrocytes and fibroblasts (11–13). The role of this protease is not limited to the reactions of innate immunity—CathG participates in the presentation of antigens and stimulation of specific immune responses (9, 10). Besides, the enzyme neutralizes toxins and has antimicrobial properties (14).

CathG activity is involved in the pathogenesis of some diseases associated with chronic inflammatory processes such as various neuropathies, atherosclerosis, chronic obstructive pulmonary disease, tumor processes and others (15–18). Functional activity of CathG paradoxically combines pro-inflammatory and anti-inflammatory properties which depend on the physiological conditions, the location of CathG secretion and the nature of the substrate. The final result of CathG-mediated proteolysis can lead to an increase in the inflammatory response or, conversely, to inflammation suppression (19). For example, elastinolytic activity of CathG promotes early atherogenesis; on the other hand, CathG inhibits the progression of atherosclerosis, destroying low density lipoproteins (LDL) (11). Multifunctional protease CathG is thought to be critically important in the maintenance of the delicate balance between tissue protection and destruction during inflammatory responses (6). Multiple studies indicate a very wide spectrum of CathG biological activity including regulatory, bactericidal, and destructive functions, which suggests the active involvement of CathG in protective and regenerative reactions.

CathG was detected extracellularly in the small intestine of patients with Crohn's disease as a result of local neutrophil degranulation (20). Recent data indicate the presence of this protease not only in the inflamed duodenal mucosa, where CathG is localized mainly in neutrophils, but also in the normal uninflamed mucous membranes (21, 22). The discovery of Paneth cells (PCs) as an alternative source of CathG biosynthesis in the duodenal mucosa indicates constitutive synthesis and secretion of the enzyme in the crypt epithelium (22). Using CathG as an example, we hypothesize that this immune protease can regulate not only inflammation, but may be involved also in normal physiological processes such as digestion, smooth muscle contraction, epithelial renewal, tissue remodeling, and others.

## Cathepsin G Biosynthesis in Duodenal Mucosa

Biosynthesis of CathG in the normal mucous membrane in duodenum was identified in some types of immune cells (intraepithelial lymphocytes, lamina propria lymphocytes, CD14-positive intestinal macrophages) and in PCs—specialized epithelial cells underlying Lieberkün crypts (22). CathG in PCs was localized in the secretory granules and secretory ducts (22). Belonging to the cell type that stands at the interface of host-microbial interaction, PCs normally secrete antimicrobial factors such as defensins, lysozyme, phospholipase 2, immunoglobulin A (IgA), etc. into a crypt lumen, which protects the intestinal stem cells from potentially dangerous damage by pathogens (23). Granule release from PCs into the lumen may occur continuously at a low rate, although diverse stimuli are known to trigger collective discharging of PCs (24). Secretory granules of human PCs also contain trypsin, a well-known digestive protease which is thought to be responsible for the maturation of antimicrobial peptide α-defensin-5 from its precursor (25). Thus, CathG is the second known serine protease that is found in PCs. According to the published data, CathG does not affect defensin processing (26) however other antimicrobial properties of CathG may indicate its role in the antibacterial protection of epithelial cells.

## Potential Substrates of Cathepsin G in Intestinal Mucosa

Since CathG has a capacity for protein processing, the enzyme is likely to be involved in the activation of biologically active molecules localized in the epithelial layer. CathG synthesis and secretion by PCs as well as observed sorption of the enzyme on the brush border (22) indicates the possibility of direct contact between CathG and its potential substrates, such as enteropeptidase zymogen (27) and components of the local intestinal renin-angiotensin system (RAS), expressed on the surface of the epithelium (28).

Enteropeptidase is a membrane protein of enterocytes which plays a key role in the digestive cascade, cleaving pancreatic trypsinogen to produce trypsin, thereby leading to the activation of other pancreatic zymogens by trypsin (29). A single chain precursor of enteropeptidase apparently undergoes proteolytic processing by unknown protease. It can be hypothesized that CathG activates enteropeptidase because CathG is the closest structural and functional analog of duodenase, a potential activator of bovine enteropeptidase (30, 31). Thus, CathG can participate in the digestive process by activating digestive proteases.

It is known that trypsin from PCs is stored as an inactive zymogen, and its proteolytic activator has not been identified (25). It is possible that CathG-activated enteropeptidase cleaves PCs-derived trypsinogen; trypsin in turn acts as prodefensin-convertase contributing to the innate defense of crypts. In this context there is a promising direction of the practically unexplored relationship of digestion and immunity where the link may be multifunctional regulatory proteases, such as CathG, the functional activity of which can affect both the digestive process and protective immune reactions. Multiple functions of various molecules are well-known: trypsin, a digestive protease, is also involved in protective reactions, as mentioned above. We can indicate other examples of the relationships between normal physiological processes and immune responses: for example, neurohormone melatonin, internal synchronizer of circadian rhythms, mediates the activation and proliferation of intestinal mucosal immune cells (32) etc.

CathG is shown to activate renin and convert angiotensin (5, 33). Consequently, CathG secreted by PCs can also affect local renin-angiotensin system in small intestine which is involved in the regulation of many functional and adaptation processes such as secretory/transport functions, smooth muscle contraction, epithelial renewal, tissue remodeling, and some others (34). Thus, the activity of CathG, synthesized by PCs, may be directly related to the normal functioning and maintenance of homeostasis in the small intestine.

## Effect of Cathepsin G on the Epithelial Layer

The effect of CathG on the epithelial layer can be either destructive or integrative, which in turn contributes to the disturbance or preservation of barrier function of the epithelium. It is assumed that the destroyed integrity of the epithelial barrier in ulcerative colitis is associated with the activation of the protease-activated receptors 4 (PAR 4) in the epithelium of large intestinal glands by neutrophilic CathG (35). Additionally, CathG from neutrophils has angiotensin-converting properties and contributes to an increase in the local angiotensin II concentration in the inflamed sites (5), which leads to apoptosis of epithelial cells and, as a result, to a violation of the epithelium barrier properties (36). On the other hand, CathG from PCs secreted within the epithelial monolayer, can regulate the function of E-cadherin and thus promote cell adhesion, and, therefore, the formation and maintenance of the intestinal barrier (37).

An enormous antigen load in the intestinal lumen stimulates chronic immune system activation in the normal gastrointestinal tract. Innate immunity cells participate in a complex regulatory system responsible for the balance between physiological and pathological inflammation, while CathG is believed to be a factor maintaining a fine balance between tissue protection and its damage in inflammation. Since pathological inflammation leads to a disruption of the epithelial barrier integrity and the development of a number of serious pathology such as inflammatory bowel disease, the role of CathG can be significant in maintenance of the normal state of the intestine.

## Discussion

**Figure 1:**
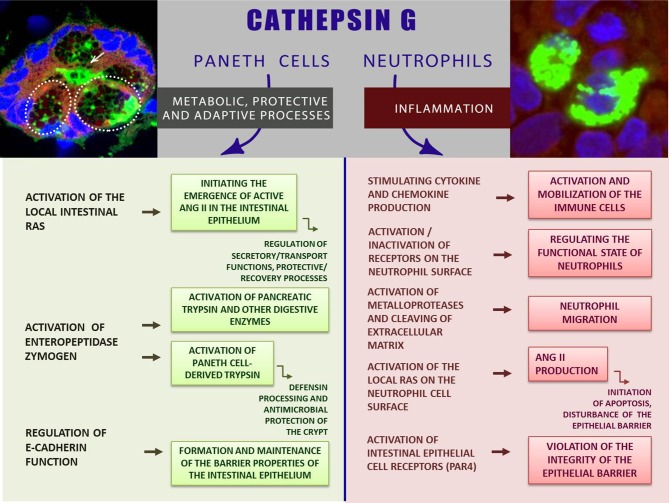
CathG—between norm and inflammation: the role of CathG in the intestinal mucosa. The inserts show CathG-specific fluorescence (green) in the intestinal gland (left); PCs are shown by dashed lines; the secretory duct is indicated by an arrow (left insert); CathG in the neutrophils (right insert). Images were obtained by immunohistochemistry and confocal microscopy [are given with modification from Zamolodchikova et al. (21, 22); relevant data are published with the permission of the Journals].

Proteases of the gastrointestinal tract are involved not only in the functioning of the digestive conveyor hydrolyzing nutrients, but also control a variety of cellular processes, providing tissue renewal and remodeling, smooth muscle contractility, hormonal regulation, and gut protection (38). In this work we aimed to focus on the role of the immune protease—CathG—in normal functioning of the intestines, meaning metabolic, protective, and adaptive processes, while the activity of this enzyme, including one in the intestinal mucosa, is traditionally considered in connection with the inflammatory process, accompanied by the migration of neutrophils synthesizing CathG. A new opinion on the role of CathG is associated with the discovery of an alternative source of CathG biosynthesis in the uninflamed intestinal mucosa—specialized epithelial cells—PCs and the secretion of this enzyme in the epithelial zone, where potential CathG substrates are localized. Available data indicate a significant biosynthesis of CathG in the normal intestinal mucosa, where neutrophils are sparse (22). We focused on the potential role of the protease in the processes associated with the intestinal functioning in order to indicate the possible role of CathG in the balance between physiological and pathological conditions of the intestinal mucous membrane. The role of CathG can be important in the development of such pathologies as inflammatory bowel diseases, the mechanisms of which are still not completely understood. The novel data concerning synthesis and secretion of CathG by PCs, as well as the presence of this enzyme in the immune cells in normal duodenal mucosa makes significant progress in understanding the role of this enzyme in the intestinal mucosa. Known more as a neutrophilic pro-inflammatory factor, CathG is also a constitutive enzyme of the normal intestinal mucosa where the protease is likely involved in the activation of proteases, receptors, and peptides in the epithelial layer, participating in the regulation of normal physiological processes, adaptive and protective functions of duodenum (Figure 1).

## Author Contributions

TZ, ST, and ES equally contributed in writing the manuscript. TZ supervised the final form.

### Conflict of Interest

The authors declare that the research was conducted in the absence of any commercial or financial relationships that could be construed as a potential conflict of interest.
